# Athena: Automated Tuning of k-mer based Genomic Error Correction Algorithms using Language Models

**DOI:** 10.1038/s41598-019-52196-4

**Published:** 2019-11-06

**Authors:** Mustafa Abdallah, Ashraf Mahgoub, Hany Ahmed, Somali Chaterji

**Affiliations:** 1 School of Electrical and Computer Engineering, Purdue University, West Lafayette, USA; 2 Department of Electronics and Electrical Communications Engineering, Cairo University, Cairo, Egypt; 3 Department of Agricultural and Biological Engineering, Purdue University, West Lafayette, USA

**Keywords:** Computational models, Computer science

## Abstract

The performance of most error-correction (EC) algorithms that operate on genomics reads is dependent on the proper choice of its configuration parameters, such as the value of *k* in *k*-mer based techniques. In this work, we target the problem of finding the best values of these configuration parameters to optimize error correction and consequently improve genome assembly. We perform this in an adaptive manner, adapted to different datasets *and* to EC tools, due to the observation that different configuration parameters are optimal for different datasets, *i*.*e*., from different platforms and species, and vary with the EC algorithm being applied. We use language modeling techniques from the Natural Language Processing (NLP) domain in our algorithmic suite, Athena, to automatically tune the performance-sensitive configuration parameters. Through the use of *N*-Gram and Recurrent Neural Network (RNN) language modeling, we validate the intuition that the EC performance can be computed quantitatively and efficiently using the “perplexity” metric, repurposed from NLP. After training the language model, we show that the perplexity metric calculated from a sample of the test (or production) data has a strong negative correlation with the quality of error correction of erroneous NGS reads. Therefore, we use the perplexity metric to guide a hill climbing-based search, converging toward the best configuration parameter value. Our approach is suitable for both *de novo* and comparative sequencing (resequencing), eliminating the need for a reference genome to serve as the ground truth. We find that Athena can automatically find the optimal value of *k* with a very high accuracy for 7 real datasets and using 3 different *k*-mer based EC algorithms, Lighter, Blue, and Racer. The inverse relation between the perplexity metric and alignment rate exists under all our tested conditions—for real and synthetic datasets, for all kinds of sequencing errors (insertion, deletion, and substitution), and for high and low error rates. The absolute value of that correlation is at least 73%. In our experiments, the best value of *k* found by **Athena** achieves an alignment rate within 0.53% of the oracle best value of *k* found through brute force searching (*i*.*e*., scanning through the entire range of *k* values). Athena’s selected value of *k* lies within the top-3 best k values using N-Gram models and the top-5 best k values using RNN models With best parameter selection by Athena, the assembly quality (NG50) is improved by a Geometric Mean of 4.72X across the 7 real datasets.

## Introduction

Rapid advances in next-generation sequencing (NGS) technologies, with the resulting drops in sequencing costs, offer unprecedented opportunities to characterize genomes across the tree-of-life. While NGS techniques allow for rapid parallel sequencing, they are more error-prone than Sanger reads and generate different error profiles, *e.g*., substitutions, insertions, and deletions. The genome analysis workflow needs to be carefully orchestrated so errors in reads are not magnified downstream. Consequently, multiple error-correction (EC) techniques have been developed for improved performance for applications ranging from *de novo* variant calling to differential expression, iterative *k*-mer selection for improved genome assembly^1^ and for long-read error correction^2,3^.

Importantly, the values of the performance-sensitive configuration parameters are dependent not only on the dataset but also on the specific EC tool (Table 2). The performance of many EC algorithms is highly dependent on the proper choice of configuration parameters, *e.g*., *k*-value (length of the substring) in *k*-spectrum-based techniques. Selecting different *k*-values has a trade-off such that small values increase the overlap probability between reads, however, an unsuitably small value degrades EC performance because it does not allow the algorithm to discern correct *k*-mers from erroneous ones. In contrast, unsuitably high *k*-values decrease the overlap probability and hurts EC performance because most *k*-mers will now appear unique. The *k*-mers that appear above a certain threshold frequency, and are therefore expected to be legitimate, are *solid k-mers*, the others are called *insolid or untrusted k-mers*. In *k*-spectrum-based methods, the goal is to convert insolid *k*-mers to solid ones with a minimum number of edit operations. Thus, an adaptive method for finding the best *k*-value and other parameters is needed for improved EC performance, and in turn, genome assembly.

Many existing EC solutions (*e.g*., Reptile^4^, Quake^5^, Lighter^6^, Blue^7^) require users to specify the *k*-mer size. Although these EC tools do not rely on a reference genome to perform correction, the best configuration value is usually found by exploration over the range of *k*-values^8^ and evaluating performance metrics, *e.g*., EC Gain, Alignment Rate, Accuracy, Recall, and Precision using a reference genome. Therefore, a reference genome is typically needed to serve as ground truth for such evaluations, making this tuning approach infeasible for *de novo* sequencing tasks. Existing tools leave the best parameter choice to the end user and this has been explicitly pointed out as an open area of work in^1,9^. However, a number of recent surveys highlighted that the automated choice of parameters for the specific dataset being processed is crucial for the user to avoid inadvertently selecting the wrong parameters^10^.

Some existing tools (e.g., KMERGENIE^11^) provide intuitive abundance histograms or heuristics to guide *k*-value selection when performing de Bruijn graph based genome assembly. However, they only account for the dataset when performing the optimal *k*-value selection. We find that this approach is unsuitable for our problem (i.e., finding best *k*-value for error correction) as the optimal *k*-value here also depends on the correction algorithm (e.g., the optimal *k*-values for Blue and Lighter in our evaluation vary, Table 2). Also, the user is finally responsible for interpreting the visualization and selecting the optimal *k*-mer value. To make the above argument specific, we found that *k* = 25 achieves within 0.04% from the best EC Gain performance for dataset *D1* when using Blue. On the other hand, if the same *k*-value was used for *D1* again but this time with the tool Lighter, the EC Gain drops by 26.8% from the maximum Gain (Tables 1 and 2 in Appendix).

In addition, there is no single *k*-value that works optimally for all datasets, even when using the *same* EC tool. For example, we found that *k* = 25 gives the best EC Gain performance for *D5* using Lighter, showing an EC Gain of 83.8%. However, the same *k*-value used for *D3* with Lighter gives an EC Gain of only 65% compared to a Gain of 95.34% when using the optimal *k*-value (Table 1 in Appendix). Thus, there is a need for a *data-driven and tool-specific* method to select the optimal *k*-value.

**Figure 1:**
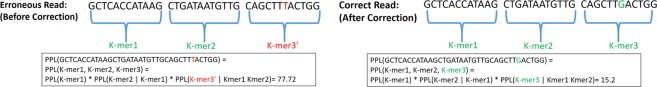
Overview of Athena’s workflow. First, we train the language model using the entire set of uncorrected reads for the specific dataset. Second, we perform error correction on a subsample from the uncorrected reads using an EC tool (*e.g*., Lighter or Blue) and a range of *k*-values. Third, we compute perplexity of each corrected sample, corrected with a specific EC tool, and decide on the best *k*-value for the next iteration, *i.e*., the one corresponding to the lowest perplexity metric because EC quality is negatively correlated with the perplexity metric. This process continues until the termination criteria are met. Finally, the complete set of reads is corrected with the best *k*-value found and then used for evaluation.

Our solution, Athena finds the best value of the configuration parameters for correcting errors in genome sequencing, such as the value of *k* in *k*-mer based methods (Just as Athena is the Greek Goddess of wisdom and a fierce warrior, we wish our technique to unearth the genomic codes underlying disease in a fearless war against maladies). Further, Athena does not require access to a reference genome to determine the optimal parameter configuration. In our evaluation, we use Bowtie2 for alignment and measure alignment rate as a metric to evaluate Athena. However, alignment is *not* needed for Athena to work as shown in Fig. 1. Athena, like other EC tools, leverages the fact that NGS reads have the property of reasonably high coverage, 30X–150X coverage depth is commonplace. From this, it follows that the likelihood of correct overlaps for a given portion of the genome will outnumber the likelihood of erroneous ones^4^. Athena uses a language model (LM) to estimate the correctness of the observed sequence considering the frequency of each subsequence and its fitness with respect to the context. This is integral to traditional NLP tasks such as speech recognition, machine translation, or text summarization in which LM is a probability distribution capturing certain characteristics of a sequence of symbols and words, which in this case is specialized to the dataset *and* to the EC tool of choice for optimal performance.

In our context, we use LM to estimate the probabilistic likelihood that some observed sequence is solid or insolid, in the context of the specific genome. We create an LM using as training, the enire original dataset (with uncorrected reads). We then run the EC algorithm on a subset of the overall data with a specific value of the configuration parameter. Subsequently, we use the trained LM at runtime to compute a metric called the “Perplexity metric” (which is widely used in the NLP literature). We show empirically that the perplexity metric has a strong negative correlation with the quality of the error correction (measured typically through the metric called “EC gain”). Crucially, the Perplexity metric evaluation does not require the computationally expensive alignment to a reference genome, even when available. Through a stochastic optimization method, we evaluate the search space to pick the best configuration-parameter value for the EC algorithm-*k* in *k*-mer-based methods and the Genome Length (GL) in the RACER EC tool. Moreover, most EC tools are evaluated based on their direct ability to reduce error rates rather than to improve genome assembly. Although in general, assembly benefits from the error correction pre-processing step, sub-optimal error correction can *reduce* assembly quality due to conversion of benign errors into damaging ones^12^. In our evaluation, we see that the EC improvement due to Athena also leads to higher quality assembly (Table 3).

In summary, this paper makes the following contributions.

1. We compare and contrast two LM variants of Athena, *N*-gram and RNN-based. Through this, we show that N-Gram modeling can be faster to train while char-RNN provides similar accuracy to *N*-gram, albeit with significantly lower memory footprint, conducive to multi-tenant analysis pipelines (*e.g*., MG-RAST for metagenomics processing^13^).
2. We introduce a likelihood-based metric, the Perplexity metric (repurposed from NLP), to evaluate EC quality without the need for a reference genome. This is the first such use of this metric in the computational genomics domain.
3. We compare and contrast two LM variants of Athena, *N*-gram and RNN-based. Through this, we show that N-Gram modeling can be faster to train while char-RNN provides similar accuracy to *N*-gram, albeit with significantly lower memory footprint, conducive to multi-tenant analysis pipelines (*e.g*., MG-RAST for metagenomics processing^13^).
4. We apply Athena to 3 *k*-mer based EC tools: Lighter^6^, Blue^7^, and RACER^14^ on 7 real datasets, with varied error rates and read lengths. We show that Athena was successful in finding either the best parameters (*k* for Lighter and Blue, and *GenomeLength* for RACER) or parameters that perform within 0.53% of the overall alignment rate to the best values using exhaustive search against a reference genome. We couple EC, with best parameter selection by Athena, to the Velvet genome assembler and find that it improves assembly quality (NG50) by a Geometric Mean of 4.72X across 7 evaluated datasets.

## Background

### Error correction and evaluation

The majority of error correction tools share the following intuition: high-fidelity sequences (or, solid sequences) can be used to correct errors in low-fidelity sequences (or, in-solid sequences). However, they vary significantly in the way they differentiate between solid and in-solid sequences. For example^4^, corrects genomic reads containing insolid *k*-mers using a minimum number of edit operations such that these reads contain only solid *k*-mers after correction. The evaluation of *de novo* sequencing techniques rely on likelihood-based metrics such as ALE^15^ and CGAL^16^, without relying on the availability of a reference genome. On the other hand, comparative sequencing or re-sequencing, such as to study structural variations among two genomes, do have reference genomes available.

### Language modeling

To increase the accuracy of detecting words in speech recognition, language modeling techniques have been used to see which word combinations have higher likelihood of occurrence than others, thus improving context-based semantics. Thus, language modeling is being used in many applications such as speech recognition^17^, text retrieval, and many NLP applications. The main task of these statistical models is to capture historical information and predict the future sequences based on that information^18^. Language models are classified into two main categories: (i) Count-based methods that represent traditional statistical models, usually involve estimating N-gram probabilities via counting and subsequent smoothing. (ii) Continuous-space language modeling is based on training deep learning algorithms. In recent years, continuous-space LMs such as fully-connected Neural Probabilistic Language Models (NPLM) and Recurrent Neural Network language models (RNNs) are proposed. Now we describe in detail each class of our language models.

### N-Gram-based language modeling

This type of modeling is word-based. The main task that N-Gram based models^19^ have been used for is to estimate the likelihood of observing a word *W*_*i*_, given the set of previous words *W*_0_, …*W*_*i*−1_, estimated using the following equation:

1 $$P({W}_{0},{W}_{1}\mathrm{,...,}{W}_{m})=\mathop{\prod }\limits_{i\mathrm{=1}}^{m}\,P({W}_{i}|{W}_{i-1}\mathrm{,...,}{W}_{1})\approx \mathop{\prod }\limits_{i\mathrm{=1}}^{m}\,P({W}_{i}|{W}_{i-1}\mathrm{,...,}{W}_{i-n})$$

where *n* represents the number of history words the model uses to predict the next word. Obviously, a higher *n* results in better prediction, at the cost of higher training time resulting from a more complex model. Also notice that for this model to operate, it has to store all conditional probability values and hence has a high memory footprint.

### Char-RNN-Based language modeling

Recurrent neural network (RNN) is a very popular class of neural networks for dealing with sequential data, frequently encountered in the NLP domain. The power of RNN is that each neuron or unit can use its internal state memory to save information from the previous input and use that state, together with the current input, to determine what the next output should be. Character-level RNN models, *char-RNN* for short, operate by taking a chunk of text and modeling the probability distribution of the next character in the sequence, given a sequence of previous characters. This then allows it to generate new text, one character at a time^20^. RNNs consist of three main layers: Input Layer, Hidden Layer, and Output Layer. First, Input Layer takes *x*_*t*_ vector, which is input at a time step *t*, usually a one-hot encoding vector of the *t*^*th*^ word or character of the input sentence. Second, Hidden Layer consists of the hidden state at the same time step *s*_*t*_, which represents the memory of this network. It is calculated as a non-linear function *f* (*e.g*., tanh) of the previous hidden state *s*_*t*−1_ and the input at current time step *x*_*t*_ with the following relation:

2 $${s}_{t}=f(U{x}_{t}+W{s}_{t-1}\mathrm{).}$$

Here, *W* is a matrix that consists of hidden weights of this hidden layer. Finally, Output Layer consists of a vector *o*_*t*_, which represents the output at step *t* and contains prediction probabilities for the next character in the sentence. Formally, its length equals the size of the vocabulary and is calculated using a softmax function. Backpropagation was used to train the RNN to update weights and minimize the error between the observed and the estimated next word. For Deep RNN architectures, there are multiple parameters that affect the performance of the model. The two main parameters are: *Number of Hidden Layers* and *Number of Neurons per Layer*. For our Char-RNN language modeling, vocabulary would include the four nucleotide bases as characters A, C, G, and T. Each input is a one-hot encoding vector for the four nucleotides. Each output vector at each time step also has the same dimension.

### Perplexity of the language model

Perplexity is a measurement of how well a language model predicts a sample. In NLP, perplexity is one of the most effective ways of evaluating the goodness of fit of a language model since a language model is a probability distribution over entire sentences of text^21^. For example, 5 per word perplexity of a model translates to the model being as confused on test data as if it had to select uniformly and independently from 5 possibilities for each word. Thus, a lower perplexity indicates that language model is better at making predictions. For an N-Gram language model, perplexity of a sentence is the inverse probability of the test set, normalized by the number of words^21^.

3 $$PP(W)=\sqrt[m]{\frac{1}{P({W}_{1},{W}_{2}\mathrm{,....,}{W}_{m})}}\approx \sqrt[m]{\frac{1}{{\prod }_{i\mathrm{=1}}^{m}P({W}_{i}|{W}_{i-1}\mathrm{,...,}{W}_{i-n})}}$$

It is clear from (3) that minimizing perplexity is the same as maximizing the probability of the observed set of *m* words from *W*_1_ to *W*_*m*_.

For RNN, perplexity is measured as the exponential of the mean of the cross-entropy loss (CE) as shown in (4)^22^, where $\hat{y}$ is the predicted next character–the output of the RNN–and |*V*| is the vocabulary size used during training.

4 $$CE(y,\hat{y})=-\mathop{\sum }\limits_{i=1}^{|V|}\,p({y}_{i})\log (p({\hat{y}}_{i}\mathrm{)).}$$

Although these two models estimate the perplexity metric differently, they achieve the same purpose, which is estimating the correctness of a sequence given the trained probability distribution. In the next section, we describe how our system Athena uses these models to find the best k-value for a given tool and dataset.

## Our Solution: Athena

### Application of language models

We use two different LM variants in our Athena algorithm. We describe them next.

#### N-Gram language models

We train an N-Gram model^19^, which is word-based, from the input set of reads before correction. This N-Gram model needs word-based segmentation of the input read as a pre-processing phase. Then, we use this trained LM to evaluate EC performance.

#### RNN language models

The second technique is RNN-based LM^23^, using different RNN architectures, *e.g*., standard RNNs, LSTMs, and GRUs. These models can be trained either as word-based models or character-based models. We train our RNN variant as character-based model to avoid having to make the decision about how to segment the genomic string, as we have to do for the N-Gram model.

#### Training time and memory footprint

Contrasting our 2 LM variants: Although training N-Gram LMs is much faster relative to RNN-based models (3–5 minutes for N-Gram *vs*. 10–11 hours for RNN-based across the 7 datasets), they still have the requirement of splitting a read into words of specific length. Further, RNN-based models have much lower memory footprint and storage requirements relative to N-Gram. This is because N-Gram models need to store conditional probabilities in large tables with an average size of 0.6–1.1 GB across the 7 datasets. In contrast, RNNs only need to store the network architecture and weights with an average size of 3–5 MB across the 7 datasets. For instance, the size of N-gram model for D7 is 2 GB while the size for the RNN model is only 3.5 MB. With respect to run time, N-gram takes 16 minutes for the whole dataset D7 while RNN takes 30 minutes on a 50 K sample of D7. Also, we used an LSTM variant of Athena and found that it took 3 times longer to train and test but gave insignificant improvement in perplexity over RNN.

### Intuition for the use of the perplexity metric

**Figure 2:**
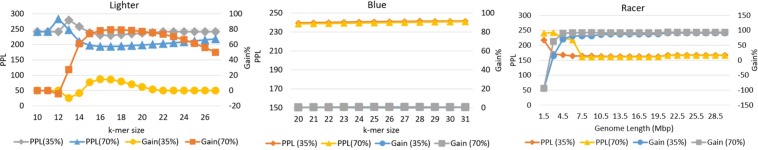
An example showing how the perplexity metric encodes errors in genomic reads. The read on the left is an erroneous read selected from dataset D3, while the read on the right is the same read, after correction with Lighter. When using language modeling to compute the perplexity for both reads, we notice that the read on the right has a lower perplexity value (15.2), relative to the erroneous read (77.72), as the sequence of *k*-mers after correction has a higher probability of occurrence. Also notice that the probability of a sequence of *k*-mers depends on both their frequencies and their relative order in the read, which allows the perplexity metric to capture how likely it is to observe this *k*-mer given the neighboring *k*-mers in a given read.

Our design uses the Perplexity metric to provide an accurate, and importantly, quick estimation of the EC quality with the current configuration parameter value(s). The Perplexity metric is based on the LM trained on the entire original (uncorrected) dataset. The Perplexity metric is then calculated on the dataset of the corrected reads (entire dataset for N-Gram and 1% for RNN) to measure the EC performance. It measures how well LM can predict the next element in an input stream. Suppose the input stream is *H* and the next element is *e*. Then, the Perplexity metric is inversely proportional to the probability of seeing “e” in the stream, given the history *H* for the learned model. Moreover, the Perplexity metric has the advantage of taking the context of the element (*e.g*., previous and subsequent *k*-mers) into consideration when estimating the probability of observing that element. This context-awareness feature is not considered in simple *k*-mer counting methods. This method works because we see empirically that there is a high negative correlation of the Perplexity metric with both EC metrics–Alignment Rate and EC Gain. Given this anti-correlation, we can rely on the Perplexity metric as an evaluation function, and apply a simple search technique (*e.g*., hill climbing) to find the best *k*-value for a given dataset. In this description, for simplicity of exposition, we use the *k*-value in *k*-mer based techniques as an example of Athena -tuned configuration parameter. However, Athena can tune any other relevant configuration parameter in EC algorithms and we experimentally show the behavior with another parameter–Genome Length–in the RACER tool. Figure 2 shows an example how Perplexity can evaluate the likelihood of a sequence of *k*-mers using their frequencies and contextual dependencies. In this example, we notice that the corrected read set (*i.e*., on the right) has a considerably lower Perplexity value (15.2), relative to the erroneous set (77.72). Thus, our intuition that the Perplexity metric reflects the correctness of the read dataset holds true here through the observed negative relationship.

### Search through the parameter space

Our objective is to find the best *k*-value that will minimize the Perplexity of the corrected dataset. The Perplexity function is denoted by *f* in (5).

5 $$\begin{array}{l}{k}_{opt}={\rm{\arg }}\,{{\rm{\min }}}_{{k}_{i}}\,Perplexity={\rm{\arg }}\,{{\rm{\min }}}_{{k}_{i}}\,f(LM,{D}_{0},{k}_{i})\end{array}$$

Here, LM: trained language model, *D*_0_: uncorrected read set, and *k*: the configuration parameter we wish to tune. *f* is a discrete function as *k*-values are discrete, and therefore, its derivative is not computable. Thus, a gradient-based optimization technique is inapplicable. Hence, we use a simple hill-climbing technique to find the value of *k* that gives the minimum value of *f*, for the given LM and *D*_0_ in (5).

The following pseudo-code describes the steps used for finding the best *k*-value for a given dataset. We start with Algorithm 1, which invokes Algorithm 2 multiple times, each time with a different starting value. We begin by training an LM on the original uncorrected read set (*D*_0_). Second, we assume that the best value of *k* lies in a range from *A* to *B* (initially set to either the tool’s recommended range, or between 1 and *L*, where *L* is the read size).

We apply an existing EC algorithm (Lighter, Blue, or RACER in our evaluation) with different initial values $({k}_{0},\ldots ,{k}_{m})\in (A,B)$ to avoid getting stuck in local minima, going through multiple iterations for a given initial value. We evaluate the Perplexity for the corrected dataset with current values of *k*: *k*_*i*_, and its neighbors, (*k*_*i*_ − *δ* and *k*_*i*_ + *δ*). Athena takes *δ* as a user input. The larger values of *δ* allows Athena to search the *k*-mers space faster, but it has the downside of missing good values of k^*^. The default value for *δ* is 1. Notice that using *δ* = 1 is not the same as applying exhaustive search to all possible values of k, as with the hill-climbing technique that Athena uses, the search is terminated when the current *k*-mer is better than its neighbours (as shown in step 2 in Algorithm 2). In each iteration, we apply hill-climbing search to identify the next best value of *k*_*i*_ for the following iteration. The algorithm terminates whenever the Perplexity relative to *k*_*i*_ is less than the perplexities of both its neighbors or the maximum number of (user-defined) iterations is reached. However, all shown results are with respect to only one initial value (*i.e*., *m* = 0 in *k*_0_, *k*_*i*_, …, *k*_*m*_).

#### Time and space complexity

Because we apply hill climbing search to find the best value of *k*, the worst-case time complexity of the proposed algorithm is *L* × |*S*′|, where *L* is the upper bound of the range of *k*-values to search for and |*S*′| is size of selected sample. For the space complexity, Athena only needs to save the Perplexity values of previously investigated *k*-values, which is also linear in terms of *L*.

**Algorithm 1:**
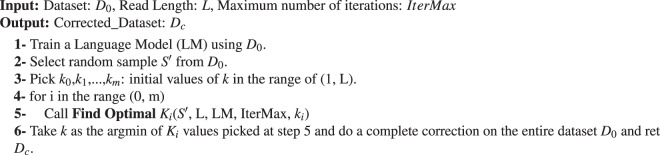
Correct Set of Reads.

**Algorithm 2:**
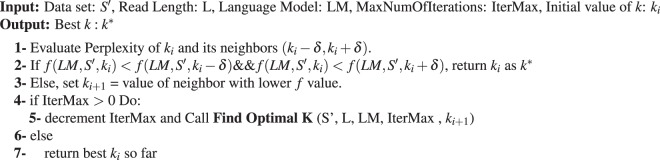
Find Optimal *k*.

## Evaluation with Real Datasets

In this section, we evaluate Athena variants separately by correcting errors in 6 real datasets and evaluating the quality of the resultant assembly.

### Implementation notes and dataset

We implement the N-Gram model using SRILM toolkit^24^. SRILM is an open source toolkit that supports building statistical N-Gram LMs, in addition to evaluating the likelihood of new sequences using the Perplexity metric. For the RNN LM implementation, we build on the TensorFlow platform^25^. Specifically, we utilized Character-level RNN models, char-RNN for short, which takes a chunk of text and models the probability distribution of the next character in the sequence, given a sequence of previous characters. After correction, we run the Bowtie2 aligner^26^ and measure the Alignment Rate and the Error Correction Gain (EC Gain). A higher value for either metric implies superior error correction. We do a sweep through a range of *k*-values and measure the alignment rate to determine if the Athena -generated *k*-value is optimal or its distance from optimality.

For interpreting the execution time results, our experiments were performed on Dell Precision T3500 Workstation, with 8 CPU cores, each running at 3.2 GHZ, 12GB RAM, and Ubuntu 16.04 Operating System. We use 3 EC tools, in pipeline mode with Athena, namely, Lighter, Blue, and RACER. Blue uses a *k*-mer consensus to target different kinds of errors, *e.g*., substitution, deletion and insertion errors, as well as uncalled bases (represented by N). This improves the performance of both alignment and assembly^7^. In contrast, Lighter is much faster as it uses only a sample of *k*-mers to perform correction. We use the automated subsampling factor (called alpha) selection option in Lighter, which is estimated based on the genome size. Third, RACER uses a different configuration parameter distinct from the *k*-value, specifically Genome Length (GL), and we are able to tune GL as well. Our ability to tune any of these EC algorithm’s parameters is in line with our vision and ongoing work to design extensible blocks of software to expedite algorithmic development in bioinformatics^27^. Incidentally, we started using another popular EC tool, Reptile, but it only allowed for a smaller range of *k*-values, beyond which it ran into out-of-memory errors. Hence, to demonstrate results with the full range of *k* values, we restricted ourselves to Lighter, Blue, and RACER. Our datasets are Illumina short reads (Table 1), used in multiple prior studies (*e.g*.^4,6,28,29^). For these, there exist ground-truth reference genomes, which we use to evaluate the EC quality. The seven datasets have different read lengths (from 36 bp to 250 bp) and different error rates (from <3% to 43%).

**Table 1:** Datasets’ description with coverage, number of reads, read lengths, genome type, and the Accesson number.

Dataset	Coverage	#Reads	Read Length	Genome Type	Accession Number
D1	80X	20.8M	136 bp	*E. coli* str. K-12 substr	SRR001665
D2	71X	7.1M	47 bp	*E. coli* str. K-12 substr	SRR022918
D3	173X	18.1M	36 bp	*Acinetobacter* sp. ADP1	SRR006332
D4	62X	3.5M	75 bp	*B. subtilis*	DRR000852
D5	166X	7.1M	100 bp	*L. interrogans C* sp. ADP1	SRR397962
D6	70X	33.6M	250 bp	*A. thaliana*	ERR2173372
D7	67X	202M	101 bp	*Homo sapiens*	SRR1658570

Coverage is estimated according to Illumina’s documentation^49^.

### Optimal parameter selection

The results of using Athena with Lighter, Blue, and RACER tools are shown in Table 2 for each dataset. The value of *k* found through exhaustive testing (or, GL for RACER) along with the EC quality is given first. Then it is noted when the Athena -selected configuration matches this theoretical best. We find that in 27 of 36 cases (75%) the configuration found by Athena matches the theoretical best. In the remaining cases, our chosen parameters are within 0.53% in overall alignment rate to the best values. We see that the optimal parameter value almost always corresponds to the lowest perplexity scores computed using Athena’s language modeling (Tables 1 and 2 in Appendix). Further, the anti-correlation between perplexity and alignment rate holds for both optimal and non-optimal *k*-values. This shows that our hypothesis is valid across a range of *k*-values. We notice that the feasible range of *k*-values in Blue is (20, 32), distinct from Lighter’s. Another interesting observation is that the optimal *k*-values are different across the two different EC tools, Lighter and Blue, for the same dataset, as observed before^6^. Athena can be applied to a different configuration parameter, GL for the RACER tool, in line with our design as a general-purpose tuning tool.

**Table 2:** Comparison of Lighter, Blue, and RACER using 7 datasets.

Dataset	Exhaustive Search			With Athena (RNN)			With Athena (N-gram)		
Lighter									
	Selected *k*	Alignment Rate (%)	EC Gain (%)	Selected *k*	Alignment Rate (%)	EC Gain (%)	Selected *k*	Alignment Rate(%)	EC Gain (%)
**D1**	**k** **=** **17**	98.95%	96.30%	Same as Exhaustive Search			Same as Exhaustive Search		
**D2**	**k** **=** **15**	61.42%	73.80%	**k** **=** **17**	61.15%	80.10%	Same as RNN		
**D3**	**k** **=** **15**	80.44%	86.78%	**k** **=** **17**	80.39%	95.34%	Same as Exhaustive Search		
**D4**	**k** **=** **17**	93.95%	89.87%	Same as Exhaustive Search			Same as Exhaustive Search		
**D5**	**k** **=** **17**	92.15%	81.70%	**k** **=** **25**	92.09%	83.80%	Same as Exhaustive Search		
**D6**	**k** **=** **25**	86.16%	**NA**	**k** **=** **17**	85.63%	**NA**	Same as RNN		
**D7**	**k** **=** **15**	40.53%	37.58%	Same as Exhaustive Search			**k** **=** **17**	40.24%	7.70%
**Blue**									
**D1**	**k** **=** **20**	99.53%	99%	**k** **=** **25**	99.29%	98.60%	Same as Exhaustive Search		
**D2**	**k** **=** **20**	57.44%	4.61%	Same as Exhaustive Search			Same as Exhaustive Search		
**D3**	**k** **=** **20**	84.17%	99.20%	Same as Exhaustive Search			Same as Exhaustive Search		
**D4**	**k** **=** **20**	95.31%	98.50%	Same as Exhaustive Search			Same as Exhaustive Search		
**D5**	**k** **=** **20**	92.33%	88.90%	Same as Exhaustive Search			Same as Exhaustive Search		
**D6**	**k** **=** **30**	86.18%	**NA**	Same as Exhaustive Search			**k** **=** **25**	86.07%	**NA**
**D7**	**k** **=** **25**	17.19%	3.57%	**k** **=** **30**	16.96%	1.47%	Same as Exhaustive Search		
**RACER**									
**D1**	**GL** **=** **4.7M**	99.26%	84.80%	Same as Exhaustive Search			Same as Exhaustive Search		
**D2**	**GL** **=** **4.7M**	81.15%	92.90%	Same as Exhaustive Search			Same as Exhaustive Search		
**D3**	**GL** **=** **3.7M**	84.11%	88.27%	Same as Exhaustive Search			Same as Exhaustive Search		
**D4**	**GL** **=** **4.2M**	95.33%	97%	Same as Exhaustive Search			Same as Exhaustive Search		
**D5**	**GL** **=** **4.2M**	92.29%	81.63%	**GL** **=** **20M**	92.28%	80.50%	Same as Exhaustive Search		
**D6**	**GL** **=** **120M**	86.36%	**NA**	Same as Exhaustive Search			**GL** **=** **20M**	86.12%	**NA**
**D7**	**GL** **=** **3M**	17.55%	21.10%	**GL** **=** **20M**	17.40%	26.50%	Same as Exhaustive Search		

This is for finding the best *k*-value (GL for RACER) using Athena variants *vs*. exhaustive search. We find either the optimal value or within 0.53% (over Alignment Rate) and within 8.5% (EC Gain) of the theoretical best (in the worst case), consistent with the reported results by Lighter (Figure 5 in^6^). These slightly sub-optimal configurations is due to the impact of sub-sampling. However, with appropriate sampling rate selection, A**thena** achieves configurations that is 0.53% of the oracle best configuration (found with exhaustive searching). We also notice that for RACER, GL found by Athena is within 3% of the reference GL (except for the RNN model with *D5*, which still achieves very close performance for both Alignment Rate and EC Gain).

The Gain metric is not shown for *D6* as the tool used to compute it was not able to handle reads of length 250 bp. We notice that the best genome length found by Athena for D7 (human genome) is 20Mbp, which is very low compared to the actual human genome length (≈3 k Mbp). This shows that using heuristics to estimate the optimal value of K based on only one parameter (genome length) can produce significantly suboptimal performance, even if the actual value of the genome length is provided. Moreover, GenomeLength parameter in Racer represents the approximate length of the DNA molecule that originated the reads. If only parts of a genome were sequenced, then only the total length of those parts should be used, instead of the length of the total genome. Dataset D7 is just for a part of the genome (24 Mbp), and Athena ‘s genome length selection of 20Mbp shows the efficacy of Athena for this usecase.

### N-gram language model results

We start by training an N-Gram language model from the original dataset. We divide each read into smaller segments (words) of length *L*_*s*_ (set to 7 by default). A careful reader may be concerned that selecting the best value of *L*_*s*_ is a difficult problem in itself, of the same order of difficulty as our original problem. Fortunately, this is *not* the case and *L*_*s*_ selection turns out to be a much simpler task. From domain knowledge, we know that if we use *L*_*s*_ = 4 or less, the frequencies of the different words will be similar as this increases the overlap probability between the generated words, thus reducing the model’s discriminatory power, while a large value will mean that the model’s memory footprint increases. We find that for a standard desktop-class machine with 32 GB of memory, *L*_*s*_ = 8 is the maximum that can be accommodated. Further, we find that the model performance is *not* very sensitive in the range (5–7), so we end up using *L*_*s*_ = 7. The same argument holds for selecting a history of words, and we use a tri-gram model (history of 3 words, i.e., n = 3) for all our experiments. Second, we compare the perplexity metric for datasets corrected with different *k*-values and compare the perplexity metric (without a reference genome) to the alignment rate (using a reference genome). We always report the *average perplexity*, which is just the total perplexity averaged across all words. Our results show a high negative correlation between the two metrics on the 7 datasets (≤−0.930 for the first six datasets and ≤−0.723 for D7), as shown in Table 3. To reiterate, the benefit of using the perplexity metric is that it can be computed without the ground truth and even where a reference genome is available, it is more computationally efficient than aligning to the reference and then computing the alignment rate.

**Table 3:** Comparison of Overall Alignment Rate of Fiona versus RACER (with and without A**thena**’s tuning).

—	Correlation to Alignment		Comparison with FIONA			Assembly quality		Runtime Improvement	
Dataset	Correlation (N-Gram)	Correlation (RNN)	Fiona + Bowtie2 (Alignment Rate)	RACER w/o Athena + Bowtie2 (Alignment Rate)	RACER w/Athena + Bowtie2 (Alignment Rate)	NG50 of Velvet w/o EC	NG50 of Velvet w/(Racer + Athena)	Athena	Bowtie2
D1	−0.977	−0.938	99.25%	85.01%	**99.26**%	3019	6827 (2.26X)	1 m 38 s	10 m 5 s
D2	−0.981	−0.969	73.75%	58.66%	**81.15**%	47	2164 (46X)	49 s	3 m 53 s
D3	−0.982	−0.968	83.12%	80.79%	**84.11**%	1042	4164 (4X)	1 m 39 s	7 m 50 s
D4	−0.946	−0.930	**95.33%**	93.86%	**95.33%**	118	858 (7.27X)	52 s	3 m 8 s
D5	−0.970	−0.962	**92.34**%	90.91%	92.29%	186	2799 (15X)	1 m 40 s	9 m 42 s
D6	−0.944	−0.979	**87.43**%	85.76%	86.84%	1098	1237 (1.12X)	6 m 40 s	1 h 42 m
D7	−0.723	−0.862	NA	17.17%	17.55%	723	754 (1.04X)	16 m	71 m

RACER requires the user to enter a value for the “Genome Length”, which has no default value. Therefore, “RACER w/o Athena” is RACER operating with a fixed Genome Length of 1M. Columns 5 & 6 demonstrate the strong anti-correlation values between Perplexity and Alignment Rate. The last two columns show the assembly quality (in terms of NG50) before and after correction by RACER, tuned with Athena. Improvements in NG50 are shown between parentheses, while NGA50 and the amount of assembly errors metrics showed similar improvements and hence omitted. We also show the search time comparison for estimating the perplexity metric with Athena (N-gram) for a point in search space *vs*. estimating overall alignment rate with Bowtie2.

### Char-RNN language model results

For training our RNN, we used the “tensorflow-char-rnn” library^25^. After parameter tuning, we use the following architecture for our experiments: 2 hidden layers with 300 neurons per layer, output layer with size 4 (*i.e*., corresponding to the four base pairs), mini-batch size 200, and learning rate 2*e*^−3^ respectively. For each of the 7 datasets, we used 90% for training and 10% for validation, with no overlap, for coming up with the optimal RNN architecture.

For our char-RNN results, we find that the perplexity metric has a strong negative relation to the overall alignment rate (Table 3), with the absolute value of the correlation always greater than 0.86. Here, we have to sample the corrected set for calculating the perplexity measure because using an RNN to perform the calculation is expensive. This is because it involves, for predicting each character, doing 4 feed-forward passes (corresponding to the one-hot encodings for A, T, G, or C), each through 600 neurons. Empirically, for a test sample size of 50 K, this translates to approximately 30 minutes on a desktop-class machine. In the experiments with the real datasets, we use 50 *K* samples with uniform sampling, and in synthetic experiments, we use 100 *K* samples (i.e., only 1% of the dataset). Importantly, the strong quantitative relationship between perplexity and the EC quality is maintained even at the low sampling rate (0.5% for real dataset). Note that this test sample size is different from the sample size used by the EC tool to perform the correction, shown in Fig. 1, which must be at least of coverage 30X to ensure accurate *k*-mer analysis^30^.

### Comparison with a self-tuning EC tool

Here, we compare Athena with the EC tool, Fiona^31^, which estimates its parameters internally. The purpose of this comparison is to show that Athena can tune *k*-mer-based approaches (RACER specifically for this experiment) to achieve comparable performance to suffix array-based approaches (*e.g*., Fiona), reducing the gap between the two approaches.

The works by^10^ and^32^ show a similar comparison between different EC approaches concluding that the automatic selection of configuration parameters, based on the datasets, is crucial for EC performance. However, they do not perform such parameter tuning automatically. Table 3 presents the overall alignment rate for our 6 evaluation datasets, calculated after doing correction by Fiona. We notice that RACER, when tuned with Athena, outperforms automatic tuning by Fiona in 3 of the 7 datasets (i.e., by about 0.01%, 7.4% and 1% on *D*1, *D*2, and *D*3 respectively), while they are equal in one dataset. Finally, Fiona is better on *D*5 by 0.05% and on *D*6 by 0.59%. Notice that Racer’s runtime is 5–6X faster compared to Fiona, which is similar to the runtimes reported in^33^. Moreover, we omit the result of Fiona with D7 as the tool took more than 6 hours without producing the corrected reads.

### Impact on assembly quality and searching time

Here we show the impact on genome assembly quality of using an EC tool tuned with Athena. We use Velvet^34^ to perform the assembly and QUAST^35^ to evaluate the assembly quality. We compare the NG50 before and after correction done by RACER using the best GL found by Athena. The results (Table 3) show a significant improvement on NG50 by 2.26X, 46X, 4X, 7.27X, 15X, 1.2X, and 1.04X respectively. For *D7*, the improvement is the lowest, since *D7* has the lowest alignment rate across all datasets. We also collect the NGA50 scores and it shows identical improvements as the NG50. These improvements are consistent with what was reported in^7,12^, which measured improvement due to the use of EC tools with manually tuned configuration parameters.

#### Search time improvement with Athena

Consider that in our problem statement, we are trying to search through a space of configuration parameters in order to optimize a metric (EC Gain or Alignment Rate). The search space can be large and since the cost of searching shows up as a runtime delay, it is important to reduce the time that it takes to evaluate that metric of each search point. Although EC tools don’t need a reference genome to operate, current state-of-the-art method use a reference genome to tune EC performance. As shown in^1,11^, the best value of configuration parameter *k* is found by iteratively picking a single *k*-value, run the EC tool with that value, then perform alignment (with one of several available tools such as Bowtie2), and finally compute the metric for that value. In contrast, with Athena, to explore one point in the search space, we run the EC algorithm with the *k*-value, and then compute the Perplexity metric, which does *not* involve the time-consuming alignment step. Here, we evaluate the relative time spent in exploring one point in the search space using Athena vis-*à*-vis the current state-of-the-art. The result is shown in Table 3. For this comparison, the alignment is done by Bowtie2^26^. We find that using the baseline approach, each step in the search takes respectively 6.2X, 4.8X, and 4.7X, 3.6X, 5.82X, 15.3X, and 4.4X longer for the 7 datasets. That is because the time taken by the LM to calculate the perplexity is linear in terms of the input (number of reads x read length), while the runtimes of alignment algorithms are superlinear^36^.

Further, while we use the hill-climbing technique to search through the space, today’s baseline methods use exhaustive search, such as in Lighter^6^ and thus the end-to-end runtime advantage of Athena will be magnified.

### Impact of sub-sampling

One drawback of sub-sampling is the reduction of coverage, which can negatively impact the accuracy of k-mer analysis. Therefore, we select the sub-sampling ratio so that the sample size has a coverage of at least 30X, which was found sufficient by several prior works (such as^30^) for accurate k-mer analysis. When the coverage is less than 30X, the distribution of solid *k*-mers *vs*. non-solid *k*-mers becomes very close, for which EC tools (*e.g*., Lighter with dataset D2) will not perform any correction for any given value of *k*. So it is a limitation in EC tools in general to require higher coverage for accurate error correction. For datasets with coverage less than 30X, we use the complete dataset without any subsampling.

We use the well-known Lander-Waterman’s formula^37^ to estimate the required sample-size to reach such coverage. If the data coverage is less than 30X, the whole dataset is used.

**Figure 3:**
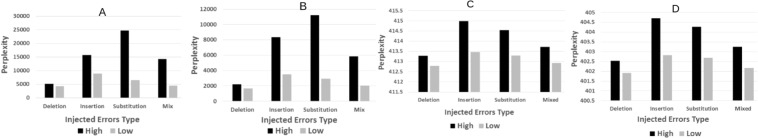
Impact of sub-sampling on perplexity and gain. We compare the perplexity and gain with samples of sizes 35% and 70% of the *D2* dataset. We observe the negative correlation between both metrics and also the positive correlation between the values of each metric on the two samples.

We also notice from Table 2 that Athena sometimes proposes slightly sub-optimal configurations compared to oracle best (within 0.53% in terms of overall alignment rate). This is due to the impact of sub-sampling as the best configuration for the sample can be slightly different from the best configuration for the complete dataset. We study the impact of sub-sampling on the accuracy of Athena. We use *D2* for this experiment as it is the one with the highest error rate across all 7 datasets. First, we select two random samples from *D2* with sampling rates of 35% (30X) and 70% (50X) respectively and investigate the correlation between perplexity and EC gain. As shown in Fig. 3, perplexity and EC gain have a very high inverse correlation, for all three tools, across the entire range of *k*-values. This shows that sampling in Athena has the advantage of significant improvements in runtime, while preserving the accuracy of optimal parameter selection.

Second, we select a random sample from *D2* with a sampling rate of 40%. We scan over the range of values of the tuning parameter (*k*-mer size for Lighter and Blue, Genome length for RACER) and calculate perplexity and EC gain for each value. We repeat this experiment with 70 different random samples and find that the selected value did not change in all runs, which also matches the best value with exhaustive searching over the complete dataset. This shows that the sub-sampling does not overfit the data and preserves the accuracy of the optimal parameter selection.

## Evaluation with Synthetically Injected Errors

Here we experiment on datasets where we synthetically inject errors of three kinds - insertion, deletion, and substitution. The real data sets used in our evaluation in Section belonged to Illumina sequencing platform and therefore had primarily substitution errors (about 99% of all errors). However, other platforms such as 454 or Ion torrent sequencing suffers primarily from insertions and deletions^10^. Hence our synthetic injections are meant to uncover if the relationship between perplexity and error rate holds for these other error types. We start by randomly collecting 100 K short reads from the reference genome (*i.e*., almost error-free) for two organisms used in the real datasets–*E. coli* (D1, D2) and *Acinetobacter* (D3). Afterward, we inject errors of each type as follows:

1. **Deletion:** We select the index of injection (position of the deleted segment), which follows a uniform distribution U(0, *L* − *d*), where *L* is the length of the read and *d* is the length to delete.
2. **Insertion:** Similar to deletion errors, the index of insertion follows a uniform distribution U(0, *L* − *I*), where *I* is the length of inserted segment. Moreover, each base pair in the inserted segment follows a uniform distribution over the four base pairs (A, C, G, or T).
3. **Substitution:** For this type of synthetic errors, we select *k* positions of substitutions following a uniform distribution U(0, *L*). Then, we substitute each base pair of the *k* positions with another base pair following a uniform distribution over the four base pairs (A, C, G, or T). Note that there is one-in-four chance that the substitution results in the same base pair.

**Figure 4:**
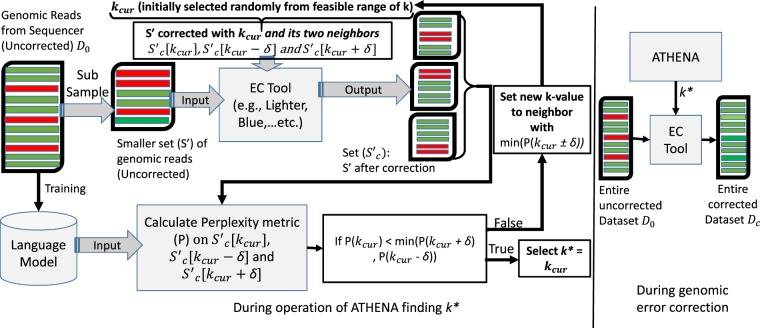
N-Gram (Figure **A**,**B**) and RNN (Figure **C**,**D**) Perplexity metric for different types of synthetic errors: Indels and Substitution errors, and a mixture of the three for *E. coli* str reference genome (Figure **A**,**C**) and *Acinetobacter* sp. reference genome (Figure **B**,**D**). We compare two versions of such errors: high and low error rates.

We test the proposed approach against two levels of synthetic errors: low error rate (Uniform in (1, 5) bp in error per read), and high error rate (Uniform in (6, 10) bp in error per read). In all cases, the language model is trained using the original data set, mentioned in Table 1, with the natural ambient rate of error. Figure 4 shows the results for the perplexity metric in the data set *after* error injection (*i.e*., without doing correction), for the three error types and an equi-probable mixture of the three. The results show that for all types of errors, the direct quantitative relation between error rate and perplexity holds–the perplexity values are higher for high error rate injection. One observation is that the values of perplexity for insertion and substitution errors are higher than for deletion errors. For example, insertion and substitution perplexities are 3X & 4.7X the deletion perplexity for high error rate data sets, and 2X & 1.5X for low error rate data sets. This is expected because in deletion errors, the segment of the read after the index of deletion is a correct segment and hence is expected by the language model. On the other hand, for insertion and substitutions, the added synthetic base pairs will most likely construct wrong segments. Such segments will have very low counts in the language model and therefore produce higher overall perplexities. Another observation is that the perplexities for the injection into data set D1 are higher than for D3. This is likely because D3 had a higher natural ambient rate of error and hence the additional injection does not cause as much increase in the perplexity metric.

## Related Work

### Error correction approaches

EC tools can be mainly divided into three categories: *k*-spectrum based, suffix tree/array-based, and multiple sequence alignment-based (MSA) methods. Each tool takes one or more configuration parameters. While we have experimented with Athena applied to the first kind, with some engineering effort, it can be applied to tuning tools that belong to the other two categories.

### Language modeling in genomics

In the genomics domain, LM was used in^38^ to find the characteristics of organisms in which N-Gram analysis was applied to 44 different bacterial and archaeal genomes and to the human genome. In subsequent work, they used N-Gram-based LM for extracting patterns from whole genome sequences. Others^39^ have used LM to enhance domain recognition in protein sequences. For example^40^, has used N-Gram analysis specifically to create a Bayesian classifier to predict the localization of a protein sequence over 10 distinct eukaryotic organisms. RNNs can be thought of as a generalization of Hidden Markov Models (HMMs) and HMMs have been applied in several studies that seek to annotate epigenomic data. For example^41^, presents a fast method using spectral learning with HMMs for annotating chromatin states in the human genome. Thus, we are seeing a steady rise in the use of ML techniques, traditionally used in NLP, being used to make sense of -omics data.

### Automatic parameter tuning

used a Feature-based Accuracy Estimator as a parameter advisor for the Opal aligner software^42,43^. The field of computer systems has had several successful solutions for automatic configuration tuning of complex software systems. Our own work^44^ plus others^45^ have shown how to do this for distributed databases, while other works have done this for distributed computing frameworks like Hadoop^46,47^ or cloud configurations^48^. We take inspiration from them but our constraints and requirements are different (such as, avoiding reliance on ground truth corrected sequences).

## Discussion

The space to search for finding the optimal configuration is non-convex in general. Therefore, it is possible to get stuck in a local minima, and hence, we use multiple random initializations. However, in our evaluation, we find that a single initialization suffices. Some EC tools have a number of performance-sensitive configuration parameters with interdependencies. There, systems such as Rafiki^44^ can encode the dependencies, while relying on Athena ‘s LM to compute the corresponding performance metric, converging toward optimal parameters. With some engineering effort, Athena can be used to optimize the *k*-value in DBG-based assemblers as well, though there will be different optimization passes since the optimal values are likely to be different for the error correction and assembly stages.

Finally, a careful reader may wonder if we can use copy number of all solid *k*-mers instead of the perplexity metric. The problem with this approach is that it will require a predefined frequency threshold to identify the solid *k*-mers. Using the perplexity metric, there is no need for such a threshold. Also the perplexity metric takes into account the context of the *k*-mer (*i.e*., previous and subsequent *k*-mers) in deciding the accuracy of the EC tool output. Also, notice that our main target is to find the optimal *k*-mer size, and different *k*-mers will have different thresholds as well.

## Conclusion

The performance of most EC tools for NGS reads is highly dependent on the proper choice of its configuration parameters, *e.g*., *k*-value selection in *k*-mer based techniques. It is computationally expensive to search through the entire range of parameters to determine the optimal value, which varies from one dataset to another. Using our Athena suite, we target the problem of automatically tuning these parameters using language modeling techniques from the NLP domain *without* the need for a ground truth genome. Through N-Gram and char-RNN language modeling, we compute the “perplexity” metric, a novel one for this problem domain, and find that the metric has a high negative correlation with the quality of genomic assembly and can be computed efficiently without using a reference genome. The perplexity metric then guides a hill climbing-based search toward the best *k*-value. We evaluate Athena with 6 different real datasets, plus with synthetically injected errors. We find that the predictive performance of the perplexity metric is maintained under all scenarios. Further, using the perplexity metric, Athena can search for and arrive at the best *k*-value, or within 0.53% of the assembly quality obtained using brute force. Athena suggests a *k*-value within the top-3 best *k*-values for N-Gram models and the top-5 best *k*-values for RNN models (the top values were determined by brute force searching).

## Supplementary information

Appendix
