# Author Correction: Athena: Automated Tuning of k-mer based Genomic Error Correction Algorithms using Language Models

**DOI:** 10.1038/s41598-020-59141-w

**Published:** 2020-02-06

**Authors:** Mustafa Abdallah, Ashraf Mahgoub, Hany Ahmed, Somali Chaterji

**Affiliations:** 1 School of Electrical and Computer Engineering, Purdue University, West Lafayette, USA; 2 Department of Electronics and Electrical Communications Engineering, Cairo University, Cairo, Egypt; 3 Department of Agricultural and Biological Engineering, Purdue University, West Lafayette, USA

Correction to: *Scientific Reports* 10.1038/s41598-019-52196-4, published online 06 November 2019

This Article contains an error in the order of the Figures. Figures 1, 2, 3 and 4 were published as Figures 4, 1, 2 and 3 respectively. The correct Figures 1, 2, 3 and 4 appear below as Figs 1–4. The Figure legends are correct.

**Figure 1:**
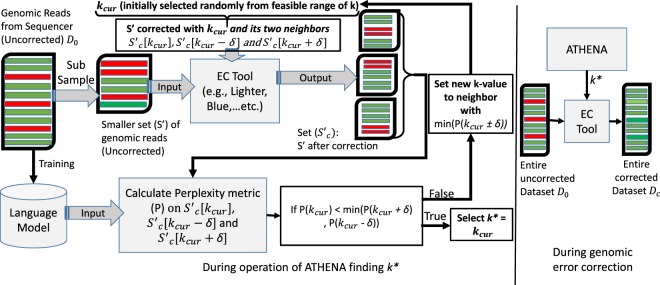
.

**Figure 2:**
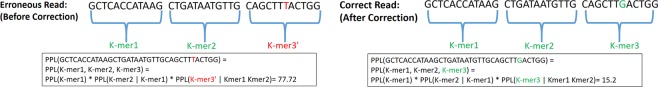
.

**Figure 3:**
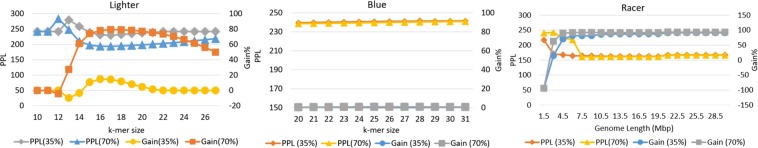
.

**Figure 4:**
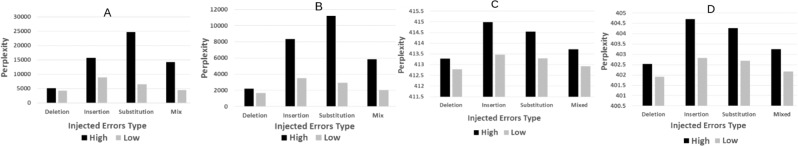
.

Additionally, the Acknowledgements section in this Article is incomplete.

“This work is supported in part by the NIH R01 Grant 1R01AI123037, a Lilly Endowment grant, a gift from Adobe Research, and funding from Purdue’s College of Engineering and Department of Ag. and Biological Engineering. Any opinions, findings, and conclusions or recommendations expressed in this paper are those of the authors and do not necessarily reflect the views of the funding agencies.”

should read:

“This work is supported in part by the NIH R01 Grant 1R01AI123037, a Lilly Endowment grant, a gift from Adobe Research, and funding from Purdue’s College of Engineering and Department of Agricultural and Biological Engineering. This material is based in part upon work supported by the National Science Foundation under Grant Numbers CCF-1919197 and CNS-1845192. Any opinions, findings, and conclusions or recommendations expressed in this paper are those of the authors and do not necessarily reflect the views of the funding agencies.”

