# Supplementary material for: Athena: Automated Tuning of k-mer based Genomic Error Correction Algorithms using Language Models
Source: Sci Rep. 2019 Nov 6;9:16157. doi: 10.1038/s41598-019-52196-4 (PMC6834855; doi:10.1038/s41598-019-52196-4)
Supplement: Supplementary file 1 — Appendix [file 41598_2019_52196_MOESM1_ESM.pdf]

ATHENA: Automated Tuning of *k*-mer based Genomic Error Correction Algorithms using Language Models

Mustafa Abdallah[1,+], Ashraf Mahgoub[1,+], Hany Ahmed[2], and Somali Chaterji[3,*]

[1]School of Electrical and Computer Engineering, Purdue University, West Lafayette, USA
[2]Department of Electronics and Electrical Communications Engineering, Cairo University, Cairo, Egypt
[3]School of Agricultural and Biological Engineering, Purdue University, West Lafayette, USA
[*]schaterji@schaterji.io
[+]these authors contributed equally to this work

ABSTRACT

The performance of most error-correction (EC) algorithms that operate on genomics reads is dependent on the proper choice of its configuration parameters, such as the value of k in k-mer based techniques. In this work, we target the problem of finding the best values of these configuration parameters to optimize error correction and consequently improve genome assembly. We perform this in an adaptive manner, adapted to different datasets *and* to EC tools, due to the observation that different configuration parameters are optimal for different datasets, *i.e.*, from different platforms and species, and vary with the EC algorithm being applied. We use language modeling techniques from the Natural Language Processing (NLP) domain in our algorithmic suite, ATHENA, to automatically tune the performance-sensitive configuration parameters. Through the use of N-Gram and Recurrent Neural Network (RNN) language modeling, we validate the intuition that the EC performance can be computed quantitatively and efficiently using the "perplexity" metric, repurposed from NLP. After training the language model, we show that the perplexity metric calculated from a sample of the test (or production) data has a strong negative correlation with the quality of error correction of erroneous NGS reads. Therefore, we use the perplexity metric to guide a hill climbing-based search, converging toward the best configuration parameter value. Our approach is suitable for both *de novo* and comparative sequencing (resequencing), eliminating the need for a reference genome to serve as the ground truth.
We find that ATHENA can automatically find the optimal value of k with a very high accuracy for 7 real datasets and using 3 different k-mer based EC algorithms, Lighter, Blue, and Racer. The inverse relation between the perplexity metric and alignment rate exists under all our tested conditions—for real and synthetic datasets, for all kinds of sequencing errors (insertion, deletion, and substitution), and for high and low error rates. The absolute value of that correlation is at least 73%. In our experiments, the best value of k found by ATHENA achieves an alignment rate within 0.53% of the oracle best value of k found through exhaustive search (*i.e.*, scanning through the entire range of k values). With best parameter selection by ATHENA, the assembly quality (NG50) is improved by a Geometric Mean of 4.72X across the 7 real datasets.

1 Appendix

Detailed Results

In this section, we show a detailed version of the results presented in Table **??**.

EC Tool	Dataset	Tuned Parameter	Perplexity (RNN)	Perplexity (N-Gram)	Overall Alignment Rate	Gain (%)
Lighter	D1	k = 10	103.0058	20.42	97.45%	-0.01%
		k = 15	103.0048	16.86	98.83%	87.50%
		**k = 17**	**103.0040**	**16.70**	**98.95**%	**96.30**%
		k = 25	103.0055	18.22	97.98%	69.50%
	D2	k = 10	204.849	121.88	56.90%	0%
		k = 15	204.775	102.13	**61.42%**	73.80%
		**k = 17**	**204.760**	**100.30**	61.15%	**80.10**%
		k = 25	204.795	107.37	59.19%	69.96%
	D3	k = 10	200.513	52.81	72.91%	0%
		k = 15	200.432	33.26	**80.44%**	86.78%
		**k = 17**	**200.432**	**32.74**	80.39%	**95.34%**
		k = 25	200.529	42.28	75.33%	65.00%
	D4	k = 10	207.295	25.53	92.14%	0%
		k = 15	206.248	18.07	93.72%	86.14%
		k = 17	**204.899**	**17.86**	**93.95**%	89.87%
		**k = 25**	206.848	18.25	93.13%	**89.90** %
	D5	k = 10	193.121	6.44	91.92%	0%
		k = 15	193.052	5.45	92.11%	73.12%
		k = 17	193.054	**5.35**	**92.15**%	81.70%
		**k = 25**	**193.052**	5.36	92.09%	**83.80**%
	D6	k = 10	199.452	638.82	85.56%	NA
		k = 15	**198.557**	571.62	84.20%	NA
		k = 17	199.245	**521.21**	85.63%	NA
		**k = 25**	199.457	521.92	**86.16%**	NA
		k = 30	199.450	527.85	86.10%	NA
	D7	k = 10	251.64	2112.4	38.06%	0%
		**k = 15**	**251.04**	1871.3	**40.53%**	**37.58**%
		k = 17	251.59	**1866.4**	40.24%	7.7%
		k = 25	251.69	1891	38.39%	-1.2%

Table 1. Detailed results for our 7 datasets using Lighter: a comparison between finding best value of *k* using **ATHENA** variants vs exhaustive searching. These results are consistent with the reported results by Lighter's authors (Figure 5 in[?]).

EC Tool	Dataset	Tuned Parameter	Perplexity (RNN)	Perplexity (N-Gram)	Overall Alignment Rate	Gain (%)
Blue	D1	**k = 20**	206.033	**16.52**	**99.53**%	**99.00%**
		k = 25	**206.026**	16.62	99.29%	98.60%
		k = 30	206.0361	16.96	98.65%	87.60%
	D2	**k = 20**	**204.846**	**119.17**	**57.44%**	**4.61%**
		k = 25	204.848	120.52	57.09%	1.70%
		k = 30	204.847	238.98	57.00%	1.24%
	D3	**k = 20**	**200.460**	**29.89**	**84.17**%	**99.20%**
		k = 25	200.490	32.39	81.62%	97.70%
		k = 30	200.510	49.22	73.84%	13.17 %
	D4	**k = 20**	**207.179**	**46.60**	**95.31%**	**98.50%**
		k = 25	207.228	47.59	94.64%	98.40%
		k = 30	207.284	48.69	93.97%	96.50%
	D5	**k = 20**	**192.804**	**15.67**	**92.33**%	88.90%
		k = 25	192.8044	15.72	92.28%	91.20%
		k = 30	192.8077	15.79	92.22%	**92.08%**
	D6	k = 20	199.939	1692.42	82.79%	NA
		k = 25	199.569	**1682.138**	86.07%	NA
		**k = 30**	**199.516**	1693.225	**86.18**%	NA
	D7	k = 20	316.24	2017.2	16.84%	**7.34%**
		**k = 25**	316.22	**2015.3**	**17.19**%	3.57%
		k = 30	**316.09**	2052.1	16.96%	1.47%

Table 2. Detailed results for our 7 datasets using Blue Error correction tool. We notice that Blue was able to achieve good correction for all datasets except D2 and D7, which had the highest error rate.

EC Tool	Dataset	Tuned Parameter	Perplexity (RNN)	Perplexity (N-Gram)	Overall Alignment Rate	Gain(%)
Racer	D1	**GL = 4.7M**	**206.0330**	**16.60**	**99.26%**	**84.80%**
		GL = 20M	206.0360	16.90	98.85%	80.30%
		GL = 30M	206.0357	16.99	98.82%	77.60%
	D2	**GL = 4.7M**	**204.7520**	**85.14**	**81.15%**	92.90%
		GL = 7M	204.7564	85.20	81.13%	**93.00%**
		GL = 30M	204.7750	88.24	79.24%	91.90%
	D3	**GL = 3.7M**	**200.4552**	**30.40**	**84.11%**	88.27%
		GL = 20M	200.4603	33.87	80.97%	80.21%
		GL = 30M	200.4626	34.46	80.79%	75.74%
	D4	**GL = 4.2M**	**206.9420**	**17.32**	**95.33%**	**97.00%**
		GL = 20M	206.9494	17.51	95.04%	96.50%
		GL = 30M	206.9489	17.53	95.01%	95.90%
	D5	**GL = 4.2M**	193.0454	**4.77**	**92.29%**	81.63%
		GL = 20M	**193.0451**	4.78	92.28%	80.50%
		GL = 30M	193.0479	4.79	92.26%	**81.90%**
	D6	GL = 20M	199.403	**236.66**	86.12%	NA
		GL = 30M	199.401	242.61	85.76%	NA
		**GL = 120M**	**199.391**	253.34	**86.36%**	NA
	D7	**GL = 3M**	251.73	**1708**	**17.55%**	21.1%
		GL = 20M	**251.65**	1751.2	17.40%	**26.5%**
		GL = 30M	251.68	1774.4	17.38%	24.4%

Table 3. Detailed results for our 7 datasets using Racer. The first row shows the results with respect to the exact genome length (*i.e.*, calculated from the reference genome).
